# No association between COMT val158met polymorphism and suicidal behavior: meta-analysis and new data

**DOI:** 10.1186/1471-244X-11-151

**Published:** 2011-09-21

**Authors:** Carlos Tovilla-Zárate, Isela Juárez-Rojop, Teresa Ramón-Frias, Mario Villar-Soto, Sherezada Pool-García, Beatriz Camarena Medellín, Alma D Genis Mendoza, Lilia López Narvaez, Nicolini Humberto

**Affiliations:** 1 Universidad Juárez Autónoma de Tabasco, División Académica Multidisciplinaria de Comalcalco, Comalcalco, Tabasco, México; 2 Universidad Juárez Autónoma de Tabasco, División Académica de Ciencias de la Salud, Villahermosa, Tabasco, México; 3 Hospital de Alta Especialidad "Gustavo A. Rovirosa P, Villahermosa, Tabasco, México; 4 Hospital General de Comalcalco Tabasco. Secretaría de Salud, Comalcalco, Tabasco, México; 5 Departamento de Genética Psiquiátrica, Instituto Nacional de Psiquiatría "Ramón de la Fuente Muñiz", México D. F., México; 6 Servicios de Atención Psiquiátrica, Secretaria de Salud. México D. F., México; 7 Hospital General de Yajalón, Yajalón, Chiapas, México

## Abstract

**Background:**

The polymorphism COMTval158met has been associated with suicidal behavior in case-control and meta-analysis studies, but results and conclusions remain controversial. The objective of this study was to examine the association between COMT val158met with suicidal behavior in a case-control study and to assess the combined evidence -this case-control study and available data from other related studies- we carried out a meta-analysis.

**Methods:**

We conducted a case-control study with 105 patients with suicide attempts and 236 controls. Subsequently, we performed a meta-analysis of published genetic association studies by searching through Medline, PubMed and Web of Science databases.

**Results:**

No significant differences were found in the distribution of alleles (χ2 = 0.33, 1 df, p = 0.56) or genotypes (χ2 = 2.36, 2 df, p = 0.26). The meta-analysis comprising 12 association studies (including the present one) showed that the risk COMTmet allele of COMTval158/met is not associated with suicidal behavior (OR: 1.09, 95% CI: 0.97-1.23), even in the absence of heterogeneity (OR: 1.09, 95% CI: 0.97-1.23).

**Conclusion:**

Our results showed no association between COMTval158/met and suicidal behavior. However, more studies are necessary to determine conclusively an association between COMT and suicidal behavior.

## Background

Suicidal behavior is a major health problem worldwide. Suicide has been suggested to involve catecholaminergic dysfunction and to have a genetic correlate. Many significant abnormalities in catecholaminergic dysfunction have been identified in suicide attempters and completers [1]. For example, high concentration of noradrenaline with decreased α2-adrenergic binding has been described in the prefrontal cortex of suicide victims [2], whereas low concentration of 3-methoxy-4-hydroxyphenyglycol, a metabolite of norepinephrine, has been observed in subjects who attempted suicide [3-5].

Several lines of evidence suggest that suicide has a genetic component [6]. Attempted and completed suicide show familial behavior. Family studies have found that those patients more likely to display suicidal behavior have parents with a history of suicidal behavioral with a heritability of about 40-50% [7-11]. A large number of studies in twins has also been reported. The pooled data from seven twin studies report concordance rates for suicide or suicide attempt of 23.5% in monozygotic twin pairs and 0.13% in dizygotic twins [12-14]. Furthermore, the classic adoption studies demonstrate higher suicide rates in the biological parents than in adoptive parents of adoptees who died by suicide [13,15]. Despite the large amount of studies conducted, the specific genes that contribute to vulnerability for suicidal behavior are unknown. One candidate gene in the study of suicidal behavior is the gene encoding the enzyme catechol-o-methyl-transferase (COMT). The biological functions of COMT make it an attractive candidate gene for suicidal behavior. COMT is a major catabolic enzyme for catecholaminergic neurotransmitters in the brain.

The COMT gene is located on the long arm of chromosome 22 at 22q11; it spans 28 kb and contains six exons. A common polymorphism of the COMT gene is the val108/158met variant (rs4680). This polymorphism is due to a G to A transition at codon 158 of the membrane bound form of COMT, which corresponds to codon 108 of the soluble form of COMT, resulting in a valine (val) to methionine (met) substitution [16,17]. COMT is one of the enzymes that degrade catecholamines, including dopamine [16,17]. The low activity COMT genotype (COMTmet/met), consisting of a met/met allele pair, yields a 3-4 fold lower enzyme activity compared to the high activity genotype (COMTval/val), which has a val/val allele pair, whereas the COMTval/met genotype produces intermediate enzyme activity [18].

To date, the polymorphism rs4680 of COMT has been associated with a number of disorders including schizophrenia [19], bipolar disorder [20], major depressive disorder [21], obsessive compulsive disorder [22], and Parkinson's disease [23]. Several studies have investigated the association between rs4680 of COMT and suicidal behavior. Other COMT polymorphisms that have been investigated are rs362204 and 2097603 [24]. Actually more than fourteen case-control studies have been reported, as well as one study in trios [25]. However, the association of COMT with suicidal behavior remains controversial. In addition, two meta-analyses have been published to date [11,26]. To explore the possibility that some of these COMT variants have susceptibility for suicidal behavior, we conducted a case-control study in a Mexican population and then we used the combined evidence to perform a meta-analysis of all the published data.

## Methods

### Case-control study

#### Samples

A total of 105 patients were consecutively recruited from the outpatient service of the General Hospital of Comalcalco in the state of Tabasco, Mexico and from the National Institute of Psychiatry Ramón de la Fuente in Mexico City. These patients had attempted suicide between January and August 2010. In addition, 236 unrelated controls were recruited for this study. All subjects signed an informed consent to participate in the study after they were given a verbal and written explanation of the research objectives. To reduce ethnic variation and stratification effects, only Mexican subjects descending from Mexican parents and grandparents participated in this study. The study was approved by the local ethics committee and performed in accordance with the ethics standards laid down in the 1975 Declaration of Helsinki.

#### Clinical evaluation

DSM-IV Axis-I and II diagnoses were made using the Structured Clinical Interview for DSM-IV in Spanish. All patients were evaluated by a trained psychiatrist or clinical psychologists with at least a master's level degree. Following the reports in the literature, we defined a suicide attempt as a self-harm behavior with at least some intent to end one's life. Subjects were excluded when the self-injury behaviors were determined to have no suicidal intention or ideation [5].

A total of 105 patients (55 males, 50 females) were included in the study. Their mean age was 30.5 (11.40) years old (range: 14-59 years). DSM-IV main lifetime diagnoses of mental disorders among the patients were as follows: schizophrenia spectrum disorders (*n *= 50), anxiety disorders (n = 35), and undiagnosed (n = 20). The mean number of suicide attempts was 2.06. The possibility of childhood abuse sufferers was not evaluated.

Control subjects consisted of 236 volunteers (132 males, 104 females); their mean age was 34.5 (10.1) years old (range: 14-51 years). They were recruited from the Blood Donor Center of the General Hospital of Comalcalco and from the general population of the Comalcalco city area in the state of Tabasco, México. Subjects were physically healthy on medical evaluation. All were of Mexican descent and none manifested psychiatric problems, as assessed in brief interviews by psychiatrists. Informed consent was obtained from each control subject.

#### COMT val108/158met (rs4680) genotyping

Genomic DNA was extracted from peripheral blood leukocytes using a modified version of the protocol by Lahiri [27]. The final volume of the PCR reaction was 5 μL and consisted of 20 ng genomic DNA, 2.5 FL TaqMan Master Mix, and 0.125 FL 20× Assay made to order. The amplification was performed in 96-well plates using the TaqMan Universal Thermal Cycling Protocol. After the PCR end-point was reached, fluorescence intensity was measured with the 7500 real-time PCR system using SDS v2.1 software (Applied Biosystems). An allelic discrimination was performed resulting in the clear identification of three genotypes for COMT Val108/158Met polymorphism. All genotyping was performed blind to patient outcome. As a quality control in our genotyping analyses we used random blind duplicates.

### Statistical Analysis

Hardy-Weinberg equilibrium was tested using Pearson's goodness-of-fit chi-squared test. Chi-squared test or Fisher's Exact test was used to compare genotype and allele frequencies between groups. The power to detect associations given the sample size was analyzed using the Quanto 1.2 software. The power of the analysis was 0.31. The level of significance was set at 0.05.

### Meta-analysis study

#### Identification and selection of publications

A literature search comprising from January to March 2011 was performed. The publications were identified using the following search terms in Medline, PubMed and Web of Science databases: "COMT and suicidal behavior", "COMT and suicide", rs4680 and suicidal behavior", rs4680 and suicide" and "COMT Val/Met and suicide". These words were combined to retrieve the summaries. The search also implicated the review of the bibliography cited at the end of various research articles to identify additional papers not covered by the electronic search of abstracts.

To be selected, the publications had to fulfill the following criteria: (1) to be published in peer-reviewed journals, (2) to be written in English, (3) to contain independent data, (4) to be case-control association studies in which the frequencies of three genotypes were clearly stated or could be calculated, and (5) the use of healthy individuals as controls. Besides, we included one article consisting only of cases, because the *n *in this study was large and raised the detection power in the meta-analysis study [28].

### Data Extraction

The following data were obtained for each of the studies: authors, year of publication, region, number of cases and controls, number of alleles, male percentage, diagnostic status, and association results. These data were not always available for all studies. In cases of missing data, we contacted the respective authors to ask for the allele frequencies that were not included in the main text of the papers. One of the studies did not include a control group [29], but we made an adjustment accordingly based on the other studies in the literature that included a control group [26]. Briefly, we calculated the weighted frequency for a particular genotype from studies that included controls and applied it to the study not including a control group. We considered the number of the "virtual" control group equal to the number of patients in a specific study. Then the hypothetical number of subjects with the particular genotype frequency was assigned in proportion to the percentage of the same genotype which was obtained from the weighted analysis [30].

The outcomes of the meta-analysis were built by taking into consideration the following categories: a) exposed sick, b) exposed not-sick, c) not-exposed sick, and d) not-exposed not-sick. The "sick" term refers to subjects exhibiting suicidal behavior and the "exposed" term to the allele of risk (COMTmet158).

### Data analysis

For the meta-analysis procedures, we used the EPIDAT 3.1 program http://dxsp.sergas.es. This software is freely available for epidemiologic analysis of tabulated data. Data was analyzed with the random-effects model following the reports in the literature [31,32]. Sample heterogeneity was analyzed with the Dersimonian and Laird's Q test. The result of the Q test was complemented with graphs to help visualize those studies that favored heterogeneity. The results of the meta-analysis are expressed as an odds ratio (OR). To address the problem of publication bias, funnel plots were calculated by the EPIDAT 3.1 software. This plotting standardizes the effect of each of the published studies on the vertical axis and its corresponding precision on the horizontal axis. Likewise, we used the Egger's test to complement the funnel plots; the Egger's test evaluates the hypothesis of absence of bias of a publication. Finally, a chi-squared (χ2) analysis was used to calculate the Hardy-Weinberg equilibrium to evaluate genotype distribution.

## Results

### Case-control study

Of the 105 suicide attempt patients, 34 (32.4%) had the COMTval/val genotype, 58 (55.2%) the COMTval/met, and 13 (12.4%) the COMTmet/met type. Genotype frequencies in the patient group satisfied the Hardy-Weinberg equilibrium (p = 0.12). In the control group, 80 individuals (33.2) presented the COMTval/val genotype, 112 (47.6%) the COMTval/met type, and 44(15.7%) the COMTmet/met type. No significant differences in genotype (χ2 = 2.36, df = 2, p = 0.26) or allele (χ2 = 0.33, df = 1, p = 0.56) frequencies were observed between patients and the control group.

### Meta-analysis study

With regard to the literature search, a total of 18 papers were identified, but only 12 were included in this meta-analysis, including our case-control study [1,5,29,33-40] (Table 1). The six excluded studies did not comply with the inclusion criteria: in two of these studies genotype frequencies could not be obtained [24,25]; other study was carried out in families [41]; in other report the sample overlapped with a previous one [26]; the fifth was a meta-analysis [11], and the sixth a review [42].

**Table 1:** Descriptive characteristics of 13 studies on the role of COMT val158/met polymorphism in suicidal behavior

Study	Sample Size n (cases-control)	Location	Diagnosis	Number of Met alleles in cases	Gender (Male/female)		Mean Age	
					
					Cases	Control	Cases	Control
Ohara 1998 [40]	12-135	Japanese	Suicide Attempt	13				
Russ 2000 [34]	51-51	Caucasians	Suicide Ideation	46	32/19	28/23	38.1	41
Nolan 2000 [37]	84-64	US and Finnish	Suicide Attempt	77	59/15	48/16	32	41
Liou 2001 [39]	62-188	Chinese, Asiatic	Suicide Attempt	29	26/36	97/91	36.7	38.6
Rujescu 2003 [38]	328-149	German, Caucasian	Suicide Attempt	159	53/96	149/179	38.6	40
Ono 2004 [1]	163-169	Japanese, Asiatic	Completed Suicide	111	112/51	114/55		46.4
Baud 2007 [36]	427-185	Switzerland and France	Suicide Attempt	388			46.0	38.7
Zalsman 2008 [35]	201-119	Caucasian-European	Suicide Attempt	220			41.6	41.2
Perroud 2010 [29]	875	France and Switzerland	Suicide Attempt	784	256/619		39.6	
Lee 2011 [5]	197-170	Korean	Suicide Attempt	121	70/127	85/85	49.3	50.7
Nedic 2011 [33]	82-311	Croatian, Caucasians	Alcohol Dependence, Suicide Attempt	108	59/23	255/58	50.46	50.70
Tovilla-Zárate 2011	105-236	Mexican	Suicide Attempt	84	55/50	132/104	30.5	34.5

The selected studies comprised a total of 2723 cases and 1886 controls. Our meta-analysis consisted of 2723 cases, 1399 more than the last meta-analysis reported in the literature [26]. We observed that in all genotyped populations, both patients and controls were in Hardy-Weinberg equilibrium (p > 0.05), excluding the controls described by Baud et al. [36] (χ2 = 6.35, p = 0.01). We explored all populations in a combined way and we still encountered them in equilibrium (p = 0.17, and p = 0.46, respectively).

**Figure 1:**
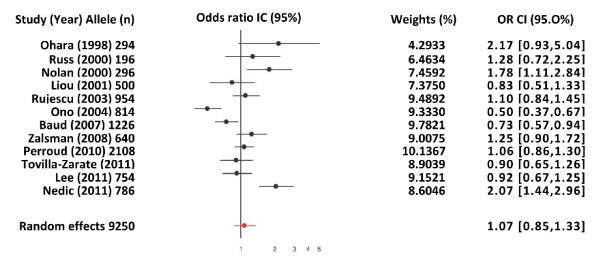
**Odds ratios for the met allele of the val/158met polymorphism in the COMT gene of individuals with suicidal behavioral**.

**Figure 2:**
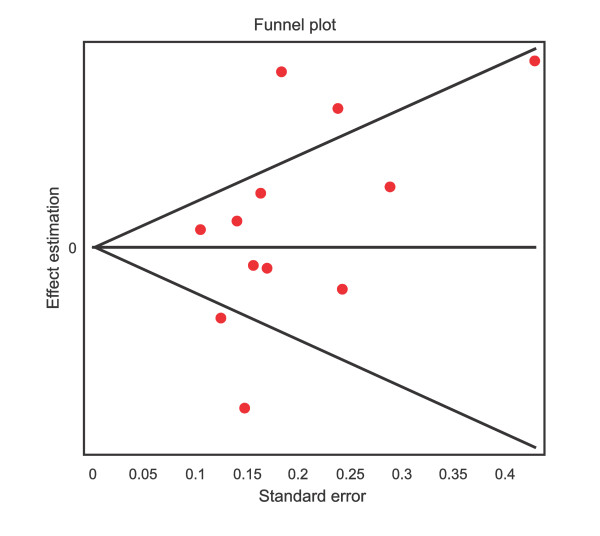
**Funnel plot indicating publication bias**.

Figure 1 shows the pooled OR derived from all studies indicating a non-significant association of allele met in the COMTval/met polymorphism with suicidal behavior (Random effects model: OR: 1.07; 95% CI 0.85-1.33; p(Z) = 0.19). We observed heterogeneity in all studies (Q = 57.08, df = 1; p = 0.0005). The Egger's test indicated no evidence of publication bias (t = 1.31, df = 10; p = 0.21) (Figure 2). Therefore, we carried out a second analysis, which only included studies inside the heterogeneity curve (Nedic [33], Ono [1], Baud [36] and Nolan [37] reports were excluded). However, we could not find an association either (OR: 1.09, 95% CI: 0.97-1.23; Z: 1.11, P(Z) = 0.26) (Table 2).

**Table 2:** Meta-analysis of case-control studies on the role of the COMT (catechol-O-methyltrasferase) val158/108Met polymorphism in suicidal behavior

References	Number of COMTval alleles		Number of COMTmet alleles		Odds Ratio (95% IC)
		
	Cases	Control	Cases	Control	
Tovilla-Zárate	126	272	84	200	0.90 (0.65-1.26)
Lee [5]	223	273	117	121	1.18 (0.86-1.61)
Perroud [29]	848	255	784	221	1.06 (0.86-1.30)
Zalsman [35]	182	121	220	117	1.25 (0.90-1.72)
Rujescu [38]	139	323	159	333	1.10 (0.84-1.95)
Liou [39]	95	275	29	101	0.83 (0.51-1.33)
Russ [34]	52	58	46	40	1.28 (0.72-2.25)
Ohara [40]	11	175	13	95	2.17 (0.93-5.04)
Random effects	1676	1752	1452	1228	1.09 (0.97-1.23)

In addition, when we included an explorative analysis of each of the Caucasian samples, significant heterogeneity was encountered (Q = 26.5; df = 5; p = 0.0001). Nedic [33] and Baud [36] reports contributed to the heterogeneity. Also, we could not detect a significant association between COMTmet allele and suicidal behavior (OR: 1.10; 95% CI: 0.91-1.16; p(Z) = 0.25).

**Figure 3:**
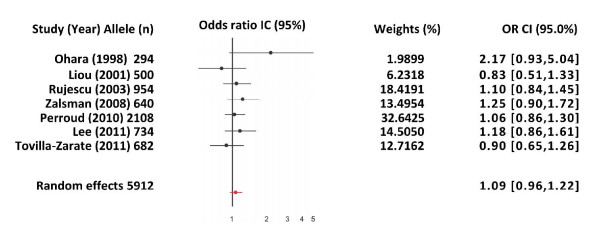
**Random-effects model, 95% CI and OR of each one of the studies with no heterogeneity and of the meta-analysis comprising the studies of the met allele of the val158met polymorphism of the COMT gene and suicidal behavior**.

Finally, we selected a subgroup of the whole sample and performed an analysis of the studies containing only suicide attempters; however, the result was also negative (OR = 1.09, 95% CI: 0.96-1.22; Z = 0.60, P(Z) = 0.54). The same occurred without the presence of heterogeneity (Q = 6.07, df = 6, p = 0.41) (Figure 3).

## Discussion

In this study, we explored the association of COMTval/met (rs4680) with suicidal behavior. First a case-control study was conducted. Additionally, we performed a meta-analysis to assess the evidence of association between COMTval/met and suicidal behavior.

We could not find any association between COMTmet or COMTval allele and suicidal behavioral in a Mexican population. To our knowledge, this is the first study addressing the genetic association between COMTval/met alleles and suicidal behavior in a Mexican population. Our results are in agreement with recent reports in the literature stating the no association of COMTval/met and suicidal behavior [26,35]. This result is not surprising considering that complex behaviors, such as suicidal behavior, are the result of a moderate number of genes that individually have small to modest effects on disease liability [26,43].

Available evidence suggests that the effect of this polymorphism on suicidal behavior may be related to the lethality of suicide attempts rather than to the risk for attempting suicide per se [11,35]. In our study we only performed an association with attempted suicide, because we wanted to establish which COMT polymorphisms were associated with suicide attempts; this could be considered as a limitation of our study. However, other study analyzing genotype differences with respect to lethality of suicide attempts or violent attempt methods reported results similar to ours [35].

It is worth mentioning the evidence provided by other studies reporting a positive association of the COMTval allele when compared to the control group [5,36-38]. But these results are controversial, since such association was observed in presence of the COMTmet allele in patients presenting alcohol dependency or other diseases [33]. These differences among studies might be explained by the different diagnostic entities used. Some studies evaluated patients with alcohol dependence, while others included patients with schizophrenia, or schizoaffective or mood disorders [33]. Other limitation could be that these association studies were conducted in various populations and different criteria were used to define the phenotypes. Other relevant factor is the difference in the size of the samples. We observed that almost all studies consisted of small samples (n < 200) and some even made subdivisions within samples (gender or affective status, for example). Hence, n was very small and had a low power of association. When we detected this limitation we decided to analyze the evidence in a meta-analysis.

We tested the probability of the association of COMTmet with suicidal behavior. However, we could not find any association between these two factors. A previous meta-analysis reported a Met association with suicidal behavior [11]; however, this association is not strong because the results of this meta-analysis were highly dependent upon the inclusion of all the studies. When five of the six studies involved were individually removed from the analysis, the relationship between COMT and suicidal behavior was no longer significant [11]. Our results are in accordance with other recently published meta-analysis in which no association was found between Met or Val allele and suicidal behavior [26]. This evidence supports a lack of direct modulation of COMT on suicidal behavior.

Similarly to the results presented in a previous study, the sample sizes of the studies included in the present meta-analysis are in the low range compared to genetics studies for other diseases. Therefore, future studies comprising larger samples of completed suicide are important to determine this association. We also consider that given the small number of studies available the association is not observable.

In a first approach we observed heterogeneity; however this variation was due to four specific studies. We carried out a second analysis, in which the studies that gave rise to heterogeneity were discarded. However, no association between COMTmet and suicidal behavior was encountered. Finally, with the aim of establishing whether this association depended on completed suicide or suicide attempt, we performed a last analysis which only included suicide attempters. Once again, no association was confirmed.

Our study presents some limitations. Our case-control study lacks of a specific scale investigating suicide attempt. With regard to the meta-analysis, publication bias has to be considered, since negative studies are less likely to get published. Also, an overrepresentation of the results showing an association between the polymorphism and the investigated disorder is also possible [44]. Although the contribution covering from genetic factors to personality traits may differ between male and female subjects, we did not analyze for gender. Other limitations are inherent in many meta-analysis of association (including this one) such as their retrospective nature and the inclusion of study-level data.

## Conclusion

In conclusion, our case-control study suggests no association between COMTmet and suicidal behavior in a Mexican population. This same negative association was observed in the meta-analysis. However, more comprehensive studies and larger samples are necessary to determine conclusively an association of COMT with suicidal behavior.

## List of abbreviations used

COMT: catechol-o-methyl-transferase; DSM-IV: Diagnostic and statistical manual of mental disorders-IV.

## Competing interests

The authors declare that they have no competing interests.

## Authors' contributions

TZC and CMB conceived the study, participated in its design, and helped to draft the manuscript. TZC, JRI, and RFT helped to perform the statistical analysis and to draft the manuscript. VSM and PGS recruited participants, and helped with the integration of data and analysis. GA, LL and HN coordinated and supervised the integration of data. All authors read and approved the final manuscript.

## Pre-publication history

The pre-publication history for this paper can be accessed here:

http://www.biomedcentral.com/1471-244X/11/151/prepub
